# Smad2/3-Regulated Expression of DLX2 Is Associated with Radiation-Induced Epithelial-Mesenchymal Transition and Radioresistance of A549 and MDA-MB-231 Human Cancer Cell Lines

**DOI:** 10.1371/journal.pone.0147343

**Published:** 2016-01-22

**Authors:** Yeo-Jin Choi, Ga-Young Baek, Hae-Ran Park, Sung-Kee Jo, Uhee Jung

**Affiliations:** 1 Radiation Biotechnology Research Division, Advanced Radiation Technology Institute, Korea Atomic Energy Research Institute (KAERI), Jeongeup, Republic of Korea; 2 Department of Radiation Biotechnology and Applied Radioisotope, University of Science and Technology (UST), Daejeon, Republic of Korea; Wayne State University School of Medicine, UNITED STATES

## Abstract

The control of radioresistance and metastatic potential of surviving cancer cells is important for improving cancer eradication by radiotheraphy. The distal-less homeobox2 (*DLX2*) gene encodes for a homeobox transcription factor involved in morphogenesis and its deregulation was found in human solid tumors and hematologic malignancies. Here we investigated the role of DLX2 in association with radiation-induced epithelial to mesenchymal transition (EMT) and stem cell-like properties and its regulation by Smad2/3 signaling in irradiated A549 and MDA-MB-231 human cancer cell lines. In irradiated A549 and MDA-MB-231 cells, EMT was induced as demonstrated by EMT marker expression, phosphorylation of Smad2/3, and migratory and invasive ability. Also, irradiated A549 and MDA-MB-231 cells showed increased cancer stem cells (CSCs) marker. Interestingly, DLX2 was overexpressed upon irradiation. Therefore, we examined the role of DLX2 in radiation-induced EMT and radioresistance. The overexpression of DLX2 alone induced EMT, migration and invasion, and CSC marker expression. The reduced colony-forming ability in irradiated cells was partially restored by DLX2 overexpression. On the other hand, the depletion of DLX2 using si-RNA abolished radiation-induced EMT, CSC marker expression, and phosphorylation of Smad2/3 in irradiated A549 and MDA-MB-231 cells. Also, depletion of DLX2 increased the radiation sensitivity in both cell lines. Moreover, knockdown of Smad2/3, a key activator of TGF-β1 pathway, abrogated the radiation-induced DLX2 expression, indicating that radiation-induced DLX2 expression is dependent on Smad2/3 signaling. These results demonstrated that DLX2 plays a crucial role in radioresistance, radiation-induced EMT and CSC marker expression, and the expression of DLX2 is regulated by Smad2/3 signaling in A549 and MDA-MB-231 cell lines.

## Introduction

Radiotherapy is one of the most common therapies for cancer, but one of its limitations is that some of surviving cancer cells can gain radioresistance [1] and metastatic ability [2] following the repeated radiotherapy. The presence of epithelial to mesenchymal transition (EMT) and cancer stem cells (CSCs) has been implicated as a putative cause of tumor radioresistance in patients [2]. CSCs are a distinct population of tumor cells with properties of self-renewal and regeneration, and have been identified in various human malignant tumors [3, 4, 5]. CSCs show typical characteristics of EMT [6, 7], and EMT is again associated with radioresistance [8, 9]. For this reason, the research and development of predictive biomarkers and targeted therapeutic strategy for CSC and EMT are especially important for prognosis evaluation and radiosensitivity improvement in radiation therapy [10, 11]. Representative CSC markers include Oct4, Sox2, Slug/Snail [12, 13, 14], and recent reports have identified particularly CD44 as a CSC-related potential biomarker for radiotheraphy in lung and breast cancer cells [15, 16, 17].

In EMT process of cancer cells, the expressions of the epithelial genes such as E-cadherin and Vinculin are inhibited, whereas the expressions of the mesenchymal genes such as N-cadherin and Vimentin are enhanced [18, 19, 20]. As a result of EMT, the cells acquire metastatic properties including loss of contact inhibition, disordered growth control, and aggressive invasiveness [18, 21]. EMT is regulated by transcription factors including Snail, Twist, and ZEB [7, 22, 23, 24]. Matrix metalloproteinases (MMPs) are also important mediators of EMT, which decompose ECM and allow the migration and invasion of cancer cells to distant sites resulting in tumor metastasis [25].

In a normal condition, TGF-β acts as a tumor suppressor but is also known to enhance EMT and to support angiogenesis in the late stage of tumorigenesis [26]. Recently, several reports showed that IR promotes EMT via Smad-dependent TGF-β signaling in cancer cell lines [6, 27, 28]. In TGF-β pathway, TGF-β receptors recognize ligands and trigger the phosphorylation of receptor-associated Smad proteins (Smad2/3) which associate with Smad4. Smad2/3/4 complexes accumulate in the nucleus and participate in the regulation of target genes expression [29].

Vertebrate distal-less homeobox2 (DLX2) acts as a homeo-box transcription factor and has crucial roles in embryonic development, craniofacial development, tissue homeostasis. According to recent reports, deregulation of DLX2 was found in human solid tumors and hematologic malignancies [30, 31, 32], and DLX2 is speculated to be involved in tumor progression via the regulation of metabolic stress-induced necrosis [33]. Moreover, DLX2 itself is a target gene of Smad-dependent TGF-β signaling and acts as a negative feedback factor of TGF-β signaling [34]. These studies made us to focus on the potential role of DLX2 in the acquirement of CSC and EMT characteristics via Smad-dependent TGF-β signaling in IR-treated cancer cells.

In this study, we have investigated the role of DLX2 in association with stem cell-like properties and epithelial to mesenchymal transition (EMT) and its regulation by Smad2/3 signaling in irradiated A549 human lung cancer cells and MDA-MB-231 human breast cancer cells. We report here that IR induced the expression of DLX2 through activation of Smad2/3, and DLX2 promoted migration and invasion, and radioresistance in A549 and MDA-MB-231 cell lines.

## Materials and Methods

### Antibodies

Antibodies against DLX2, Smad2/3, CD44, β-catenin, N-cadherin and E-cadherin were purchased from Thermo Scientific (Rockford, IL, USA). Anti-Snail, anti-Vimentin and anti-Vinculin antibodies were purchased from Cell Signaling Technologies (Danvers, MA, USA). An anti-pSmad2/3 antibody was purchased from Kerafast, Inc. (Boston, MA, USA). Anti-β-actin antibody was purchased from Santa Cruz Biotechnology (Santa Cruz, CA, USA).

### Cell culture and Irradiation

A549 (human non-small cell lung cancer cell line) and MDA-MB-231 (human breast cancer cell line) were purchased from Korean Cell Line Bank (Seoul, Korea). Cells were maintained in RPMI1640 supplemented with 10% fetal bovine serum (FBS; Hyclone, UT, USA), 100 U/ml penicillin, 100 μg/ml streptomycin at 37°C in a humidified 5% CO_2_ atmosphere. Cells were detached from the culture dish using 0.25% trypsin and diluted to 1.5 × 10^5^ cell/ml. Cells were irradiated with ^137^Cs γ-rays using a Gammacell 40 Exactor (MDS Nordion, Ontario, Canada) at the KAERI and then re-plated in culture dishes or chambers.

### Construction of the DLX2 overexpression vector and transfection

An insert of human DLX2 was amplified from pCMV-sports6/DLX2 (Korea Human Gene Bank, Seoul, Korea) by PCR using the following primers: *EcoRI* (forward): 5'-CGGAATTC ATGACTGGAGTCTTTGAC-3' and *Xho*I (reverse): 5'-CTCTGAGT TTAGAAAATCGTCCCCG-3'. DLX2 inserts was cloned in the vector pcDNA3-myc which allows expression of c-myc-tagged protein in mammalian cells. pcDNA3-myc/DLX2 or pcDNA3-myc was then delivered to cells (4 μg/2.5 × 10^5^ cells) using HilyMax (Dojindo Molecular Technologies, Rockville, ML, USA) transfection reagent according to the manufacturer’s instruction. After incubation for 24 h, the cells were detached and irradiated.

### Small-interfering RNA (siRNA) transfection

The si-RNAs targeting DLX2 and Smad2/3 were purchased from Santa Cruz Biotechnology (Santa Cruz, CA, USA). For transfection, cells were plated and grown to 70–90% confluence. Target si-RNA or negative control si-Ct was then delivered to cells (si-DLX2: 50 μM/2.5×10^5^ cells; si-Smad2/3: 100 μM/2.5×10^5^ cells) using HilyMax transfection reagent according to the manufacturer’s instruction. After incubation for 24 h, the cells were detached and irradiated.

### RNA extraction and Quantitative real-time PCR

After 24 h irradiation, total RNA extracts (1×10^6^ cells) were isolated by Trizol Reagent (Ambion, Carlsbad, CA, USA) according to manufacturer’s protocol. Reverse transcription was performed for cDNA synthesis using TOPscript RT DryMIX containing reverse transcriptase, RT buffer, dNTP mixture, Oligo dT (Enzynomics, Seoul, Korea). Quantitative real-time PCR was performed by a StepOne Real-Time PCR (Applied Biosystems, CA, USA) with SYBR Green reagent (Enzynomics, Seoul, Korea). Primers were designed using Primer-BLAST (http://www.ncbi.nlm.nih.gov/tools/primer-blast/) and the sequences are presented in Table 1. Total reaction volume of PCR mixture was 20 μl, and reaction conditions were 15 min of initial denaturation at 95°C and 40 cycles of 10 sec at 95°C, 15 sec at 60°C and 20 sec at 72°C. The comparative C_t_ method was used and relative mRNA expression level was calculated based on normalization to β-actin. All experiments were repeated in three times independently.

**Table 1:** Primer sequence for real-time quantitative PCR.

Primer	Sequences
DLX2	
Forward	5'-GCACATGGGTTCCTACCAGT-3'
Reverse	5'-ACTTTCTTTGGCTTCCCGTT-3'
OCT4	
Forward	5'-AGCAAAACCCGGAGGAGT-3'
Reverse	5'-CCACATCGGCCTGTGTATATC-3'
SOX2	
Forward	5'-GTGAGCGCCCTGCAGTACAA-3'
Reverse	5'-GCGAGTAGGACATGCTGTAGGTG-3'
LIF	
Forward	5'-GTTCCCCAACAACCTGGACA-3'
Reverse	5'-ACGACTATGCGGTACAGCTC-3'
TWIST	
Forward	5'-CTCGGACAAGCTGAGCAAGA-3'
Reverse	5'-TTGCCATCTTGGAGTCCAGC-3'
SNAIL	
Forward	5'-TTTCCTCGTCAGGAAGCCCTC-3'
Reverse	5'-TGCTGGAAGGTAAACTCTGGATTAG-3'
MMP2	
Forward	5'-GGAAAGCCAGGATCCATTTT-3'
Reverse	5'-ATGCCGCCTTTAACTGGAG-3'
MMP7	
Forward	5'-GTCACTTCTTCGGTTGTAGGGA-3'
Reverse	5'-TCAGAGGAATGTCCCATACCCA-3'
β-actin	
Forward	5'-GACCTGTACGCCAACACAGT-3'
Reverse	5'-CCAGGGCAGTGATCTCCTTC-3'

### Clonogenic assay

Detached cells were exposed to various doses of irradiation and incubated for 7 days (A549) and 10 days (MDA-MB-231) at 37°C. The medium of all cultures was renewed every 3 days. The colonies were fixed with 60% methanol and stained with 0.5% crystal violet. Colonies containing 50 cells or more were counted as clonogenic cells. The reported survival fraction values are the mean of six replicates from three independent experiments.

### Cell migration assay

To measure their migration, irradiated A549 and MDA-MB-231 cells were seeded in a transwell (Corning Incorporated, NY, USA) at a density of 2.5 × 10^4^ cells/well in 200 μl of serum-free medium and incubated for 7 h (A549) or 3 h (MDA-MB-231) at 37°C. After incubation, the medium was removed. Cells were fixed through incubation with 100% methanol and stained with 0.5% crystal violet for 15 min. The membrane was cut away from each chamber and migrated cells on the lower surface of the filter were counted per filter in random microscopic filed at a 200-fold magnification (Leica, Heidelberg, Germany). The reported values are the mean of three independent experiments.

### Cell invasion assay

The ability of irradiated A549 and MDA-MB-231 cells to pass through matrigel-coated filters was measured in a Boyden chamber invasion assay. Matrigel was applied to the top a polycarbonate filter (pore size, 8 μm). Cell invasion assays were performed using a matrigel invasion assay kit (BD Biosciences, Bedford, MA, USA) according to the manufacturer’s instructions. Briefly, cells were seeded in the upper chamber at a density of 2.5–5×10^4^ cells/well in 500 μl of serum-free medium and incubated for 48 h (A549), 24 h (MDA-MB-231) at 37°C. Cells that invaded the lower surface of each membrane were fixed with 100% methanol and stained with 0.5% crystal violet for 15 min. The membrane was cut away from each chamber and invaded cells on the lower surface of the filter were counted per filter in random microscopic filed at a 100-fold magnification (Leica, Heidelberg, Germany). The reported values are the mean of three independent experiments.

### Western blot analysis

After 24 h irradiation, total cell lysates (2×10^6^ cells) were prepared using RIPA lysis buffer (Thermo Scientific, Rockford, IL, USA) containing 10 mM phenylmethanesulphonylfluoride (PMSF), 10 mM sodium fluoride (NaF), 1 mM sodium orthovanadate (Na_3_VO_4_) and a complete protease inhibitor cocktail (Sigma-Aldrich, St. Louis, MO, USA) and the protein content was measured using the BCA protein assay reagent (Thermo Scientific, Rockford, IL, USA), with bovine serum albumin as the standard. Equal amounts of protein were resolved by 10% SDS–PAGE and transferred onto a polyvinylidene difluoride membrane (Amersham Hybond, Freiburg, Germany). The membrane was then washed with Tris-buffered saline (10 mM Tris and 150 mM NaCl) containing 0.1% Tween 20 (TBST), and blocked with TBST containing 5% nonfat dry milk powder. The membrane was incubated overnight with primary antibody and the blot was exposed to horseradish peroxidase-conjugated secondary antibody and developed using ECL Western blot detection reagents (Millipore Corporation, Billerica, MA, USA).

### Immunofluorescence (IF) staining

To analyze the intracellular localization of F-actin and marker proteins, A549 and MDA-MB-231 cells were seed in Lab-Tek chamber slide 8 well glass slide (Nunc, NY, USA) and transfection with si-Ct or si-DLX2 for 24 h and then incubation for 24 h after IR. Cells fixed in 4% formaldehyde were stained through incubation with primary antibody (rabbit polyclonal anti-N-cadherin/Vimentin/E-cadherin/Vinculin antibody, diluted 1:100) for overnight at 4°C. They were then rinsed with PBS, incubated with blocking buffer, and treated with an Alexa 546-conjugated anti-rabbit antibody (Molecular Probes, CA, USA) for 3 h at room temperature. Finally, intracellular F-actin was stained using Alexa-phalloidin-488 (diluted 1:300, Molecular Probes, CA, USA) for 1 h. The nucleus of the cell was stained with TO-PRO-3 (Molecular Probes, Eugene, USA). Stained cells were mounted and imaged using a laser confocal scanning microscope (Leica, Heidelberg, Germany).

### Determination of TGF-β1 in cell culture supernatants

A549 and MDA-MB-231 (5×10^5^ cells/well) cells were exposed to IR at 8Gy, 4Gy and incubated at 37°C for 24 h. The level of TGF-β1 protein in culture supernatants was measured using a TGF-β1 ELISA kit (BD Biosciences, San Diego, USA) according to the manufacturer's instructions. The absorbance at 450 nm was measured using a microplate reader (Molecular Devices, Sunnyvale, CA). The TGF-β1 protein levels were determined from three different experiments and are expressed as pg/ml.

### Statistical analysis

All experiments were performed three times. Statistically significant differences were identified using Student’s t-test. Statistical probability of P<0.05 was considered significant. Data represent the mean ±S.E.M. of the three experiments, each performed in triplicate.

## Results

### Ionizing radiation induces EMT and increases CD44 and DLX2 expression

**Fig 1:**
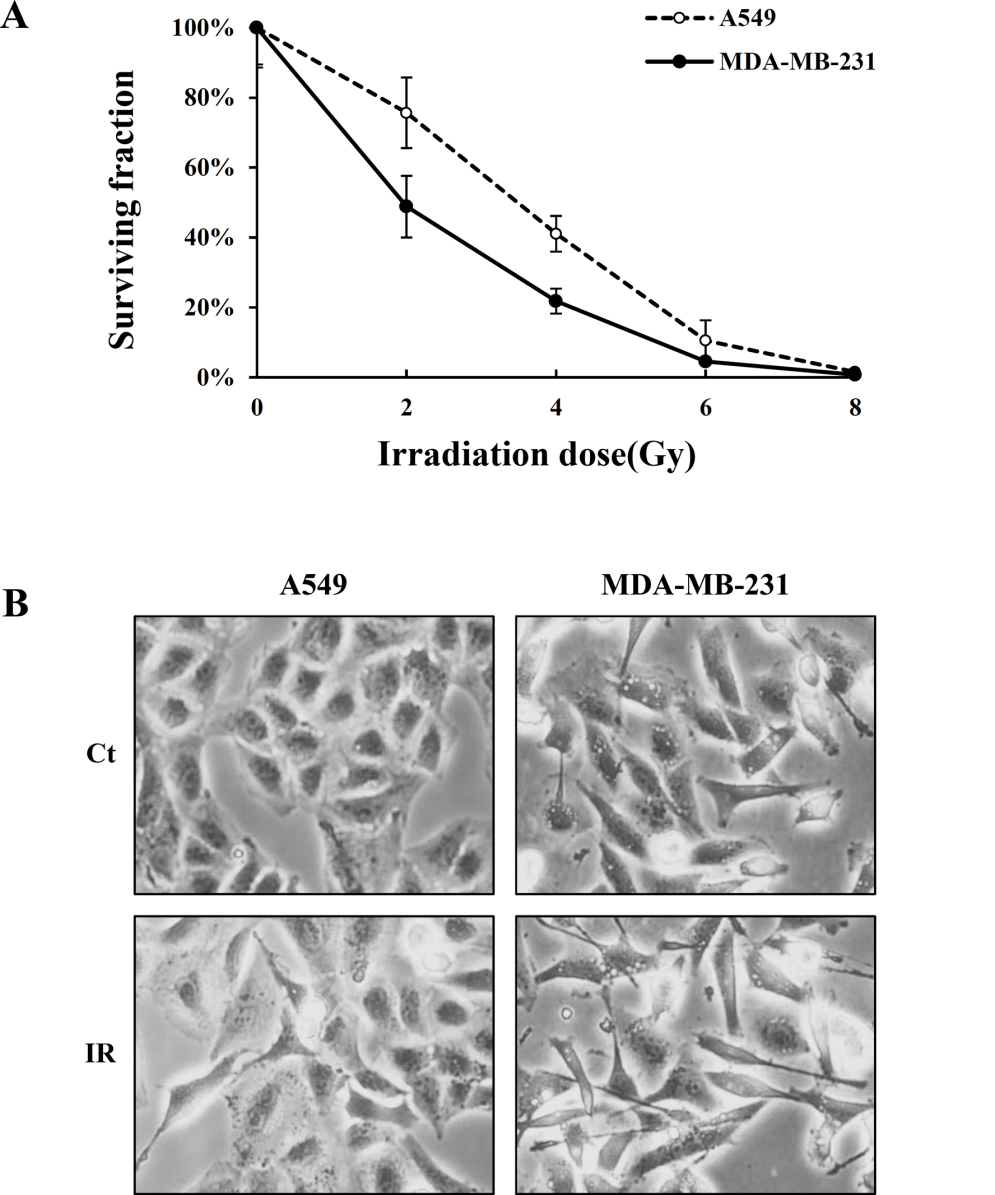
Survival curves and morphological change of A549 and MDA-MB-231 cells by ionizing radiation. (A) Detached-cells (1×10^5^cells/ml) were exposed to various doses of irradiation and incubated for 7 days (A549) and 10 days (MDA-MB-231) at 37°C. Colonies containing 50 cells or more were counted as clonogenic cells. The surviving fraction was calculated by dividing the plating efficiency of treated sample with the plating efficiency of control. Plating efficiency was calculated by dividing the number of colonies with the number of cells plated. The reported values are the mean of six replicates from three independent experiments. (B) IR induces morphological change in A549 and MDA-MB-231 cells after 24 h of exposure (A549-8Gy, MDA-MB-231-4Gy). The magnification of the image is ×100. Ct, control cell; IR, irradiated cells.

We first analyzed the radiation sensitivity of human A549 lung carcinoma and MDA-MB-231 breast adenocarcinoma cells by clonogenic assay. In this assay, cell survival was dose-dependently decreased by IR (0, 2, 4, 6, 8Gy) in both cell lines, but MDA-MB-231 cells were more sensitive to IR than A549 cells (Fig 1A). The morphological changes of cells following exposure to IR were verified in A549 cells irradiated at 8Gy and MDA-MB-231 cells at 4Gy. The irradiated A549 and MDA-MB-231cells showed spindle-shaped and elongated morphologies, which are typical of EMT morphological phenotypes when compared with the control cells (Fig 1B).

**Fig 2:**
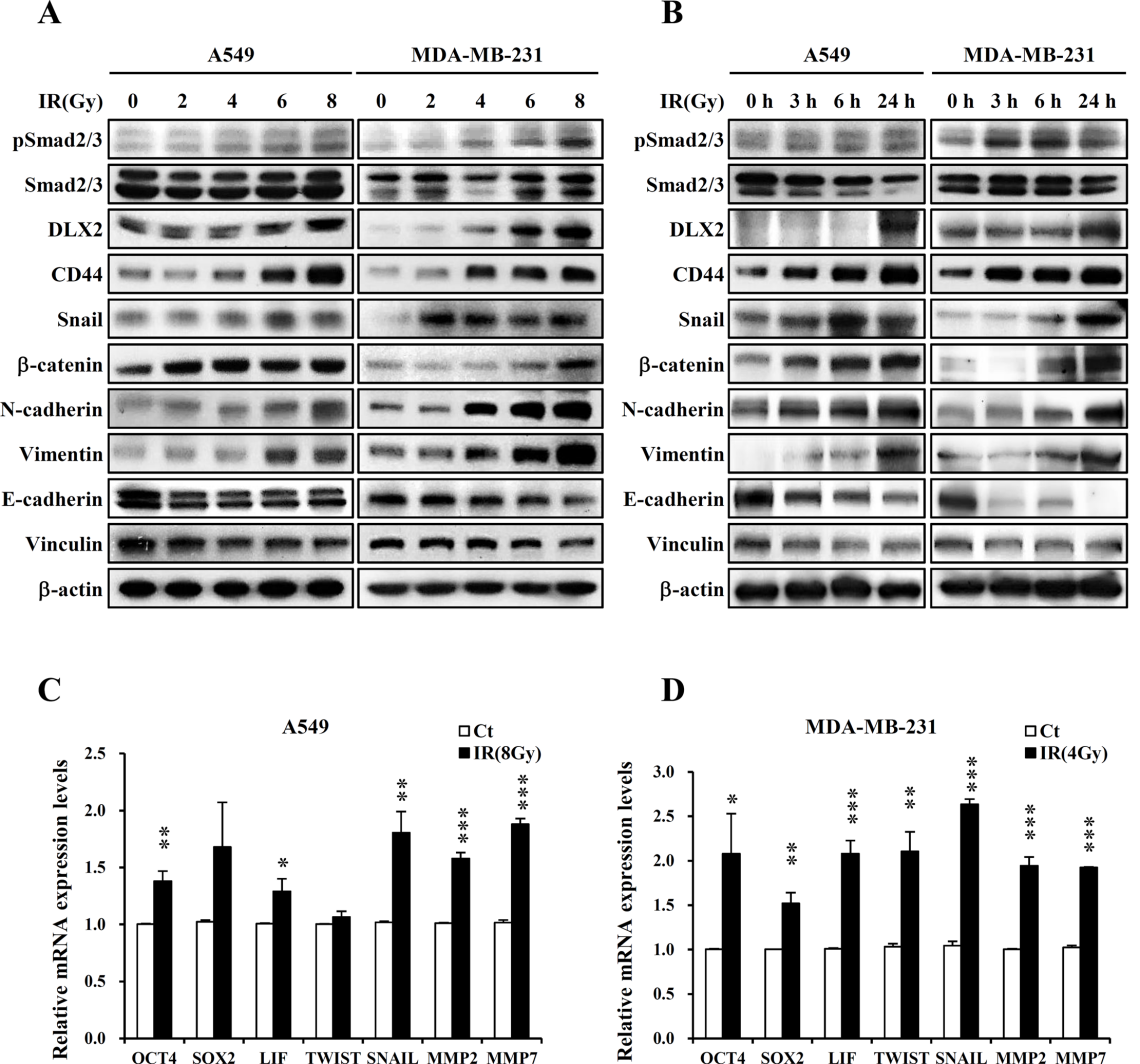
Ionizing radiation increases the expression of DLX2 and CSC markers, and induces EMT. (A) A549 and MDA-MB-231 cells were exposed to IR at 0-8Gy and incubated at 37°C for 24 h. Lysates were subjected to western blot analysis with the labeled antibodies. Two independent experiments obtained similar results. (B) A549 and MDA-MB-231 cells were harvested on 0/3/6/24 h after 8Gy (A549) or 4Gy (MDA-MB-231). Lysates were subjected to western blot analysis with the labeled antibodies. Two independent experiments obtained similar results. (C), (D) A549 and MDA-MB-231 cells were exposed to IR at 8Gy (A549) or 4Gy (MDA-MB-231) and incubated at 37°C for 24 h. Subsequently, cells were isolated by Trizol and subjected to real time PCR analysis with the labeled primers. All results were obtained from three independent experiments (***P < 0.001, **P < 0.01, *P < 0.05 versus Ct). Ct, control cell; IR, irradiated cells.

We next investigated the dose-and time-dependent changes of CSC- and EMT-related marker expression. In consistency with the previous studies, IR increased the expression of TGF-β signaling factor pSmad2/3, a CSC marker (CD44), EMT positive markers (N-cadherin, Vimentin), and transcription factors regulating EMT (Snail, β-catenin), and decreased the expression of EMT negative markers (E-cadherin, Vinculin) dependently on IR-dose or time in both cell lines (Fig 2A and 2B). Interestingly, the expression of DLX2 was also increased by IR in both cell lines. Dose–response experiments revealed that DLX2 induction was observed at 8Gy in A549 and at 4Gy or higher in MDA-MB-231 (Fig 2A and S1 Fig), and time-course experiments showed that DLX2 protein levels were dramatically increased at 24 h after irradiation in both cell lines (Fig 2B and S2 Fig). Also, IR triggered upregulation of mRNA levels of stemness markers (OCT4, SOX2, LIF), transcription factors (Twist, Snail), and metastasis markers (MMP2, MMP7) in both cell lines at 24 h after irradiation by a single dose of 8Gy for A549 cells or 4Gy for MDA-MB-231 cells (Fig 2C and 2D).

### Overexpression of DLX2 enhances EMT and radioresistance

**Fig 3:**
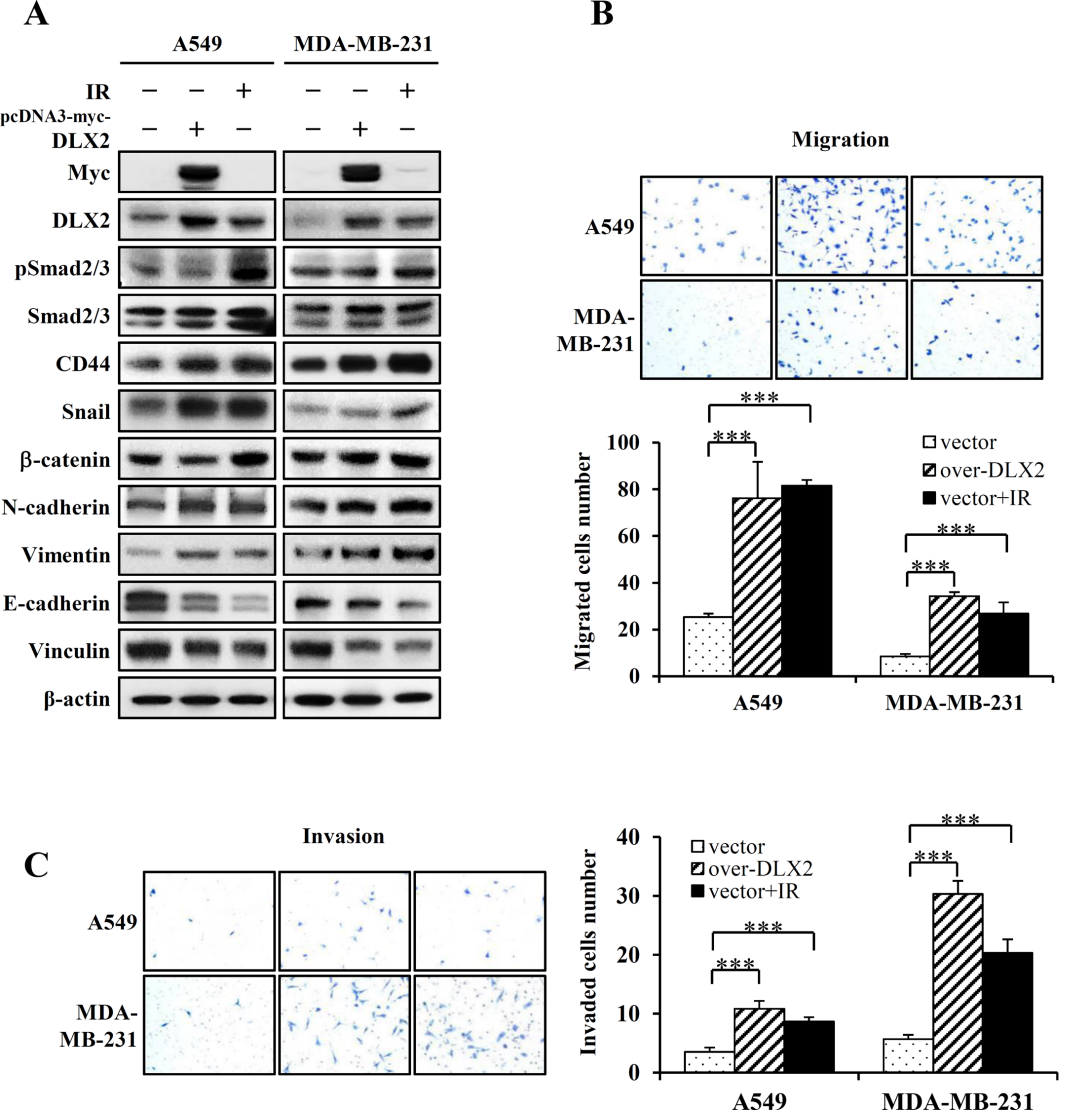
DLX2 overexpression and irradiation increases the expression of CSC markers, and induces EMT and metastatic ability in A549 and MDA-MB-231 cells. (A) Cells were transfected with 4 μg pcDNA-myc/DLX2 or pcDNA-myc and incubated at 37°C for 24 h. After cell detachment, the cells were exposed to IR at 8Gy (A549) or 4Gy (MDA-MB-231) and incubated at 37°C for 24 h. Subsequently, cells were lysed and the lysates were subjected to western blot analysis. Two independent experiments obtained similar results. (B) Cells were transfected with 4 μg pcDNA-myc/DLX2 or pcDNA-myc and incubated at 37°C for 24 h. After cell detachment, the cells were exposed to IR at 8Gy (A549) or 4Gy (MDA-MB-231) and incubated at 37°C for 6 h (A549), 3 h (MDA-MB-231), in transwell (migration assay). The photographs are representative fields of migrated cells on the membrane. The graph indicates average of migrated cell number from three independent experiments ±SE (***P < 0.001 vs. the vector control cells). (C) Cells were transfected with 4 μg pcDNA-myc/DLX2 or pcDNA-myc and incubated at 37°C for 24 h. After cell detachment, the cells were exposed to IR at 8Gy (A549) or 4Gy (MDA-MB-231) and incubated at 37°C for 48 h (A549), 24 h (MDA-MB-231), in matrigel (invasion assay). The photographs are representative fields of invaded cells on the membrane. The graph indicates average of invaded cell number from three independent experiments ±SE (***P < 0.001 vs. the vector control cells).

To examine whether DLX2 can upregulate radiation resistance and metastatic potential, A549 and MDA-MB-231 cells were transiently transfected with pcDNA3-myc vector expressing DLX2 or pcDNA3-myc control vector, and we examined the expression of CSC and EMT markers by western blot analysis. Overexpression of DLX2 increased the expression of a CSC marker (CD44) and EMT positive markers (N-cadherin, Vimentin) and decreased the expression of EMT negative markers (E-cadherin, Vinculin) in both cell lines. Irradiation induced similar changes of these markers as DLX2 overexpression. However, phosphorylation of Smad2/3 was slightly decreased (A549) or not changed (MDA-MB-231) by overexpressed DLX2 but increased by irradiation in both cell lines. Overexpression of DLX2 increased expression of Snail in A549 but not in MDA-MB-231 cells. The expression of β-catenin was not altered by DLX2 overexpression in both cell lines (Fig 3A).

To investigate the metastatic effect of DLX2 and IR in A549 and MDA-MB-231 cells, migration and invasion assays were performed. DLX2-overexpressed A549 and MDA-MB-231 cells exhibited enhanced migration ability by 3 and 4 folds, respectively, compared with the vector control (Fig 3B). Also, the exposure to IR (8Gy for A549, 4Gy for MDA-MB-231) increased migrating cell numbers by 3.2 folds in both cell lines (Fig 3B). These results showed that DLX2 overexpression and IR significantly enhanced the cell motility of A549 and MDA-MB-231 cells. To examine whether cell invasion ability was similarly enhanced by IR or DLX2 overexpression, A549 and MDA-MB-231 cells were applied to invasion chambers and numbers of adhesive cells were counted. DLX2-overexpressed A549 and MDA-MB-231 cells exhibited enhanced invasive ability by 3 and 5.3 folds, respectively (Fig 3C). Additionally, the exposure to IR increased the invading cell numbers of A549 and MDA-MB-231 cells by 2.5 and 3.6 folds, respectively (Fig 3C). These results demonstrate that overexpression of DLX2 or IR significantly enhanced the migratory and invasive ability as well as the expression changes of CSC and EMT-related genes in A549 and MDA-MB-231 cells.

**Fig 4:**
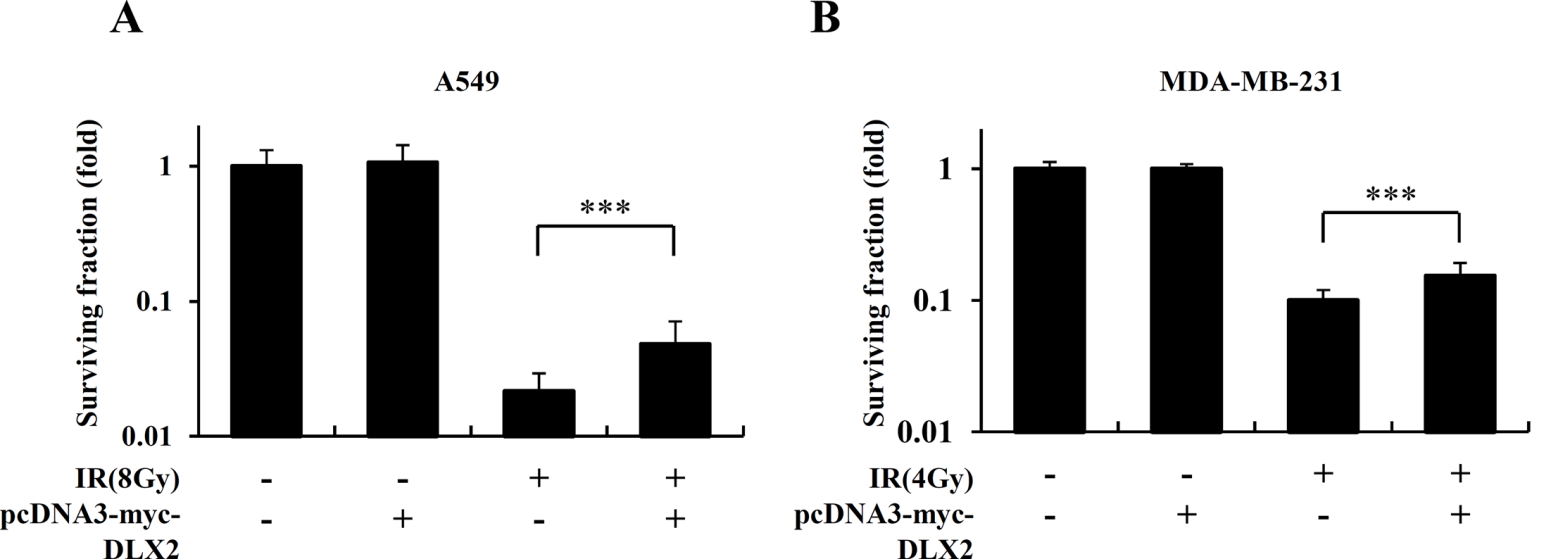
DLX2-overexpression increases cell survival in irradiated A549 and MDA-MB-231 cells. Cells were transfected with 4 μg pcDNA-myc/DLX2 or pcDNA-myc and incubated at 37°C for 24 h. After cell detachment, the cells were exposed to IR at 8Gy (A549) or 4Gy (MDA-MB-231) and incubated at 37°C for 7 days (A549), 10 days (MDA-MB-231) and cell clonogenicity measured by clonogenic assay in A549 (A) and MDA-MB-231 (B). The photographs are representative plate of survival cells. The graph represents the average of three independent experiments ±SE (***P < 0.001 vs. the IR exposed cells).

We determined next whether DLX2 would provide increased cancer cell survival after irradiation. DLX2-overexpressing A549 and MDA-MB-231 cells were irradiated at 8Gy and 4Gy, respectively, and then clonogenic assay was performed. The surviving fraction of cells transfected with control vector declined after irradiation compared to non-irradiated cells. However, DLX2-overexpressing cells showed significantly higher survival rate compared to vector-transfected cells after irradiation (Fig 4A and 4B). In non-irradiated cells, colony formation was not affected by DLX2-overexpression (Fig 4A and 4B). These results indicate that DLX2-overexpression at least partially contributes to radioresistance in A549 and MDA-MB-231 cells.

### Silencing of DLX2 inhibits IR-induced EMT and radioresistance

**Fig 5:**
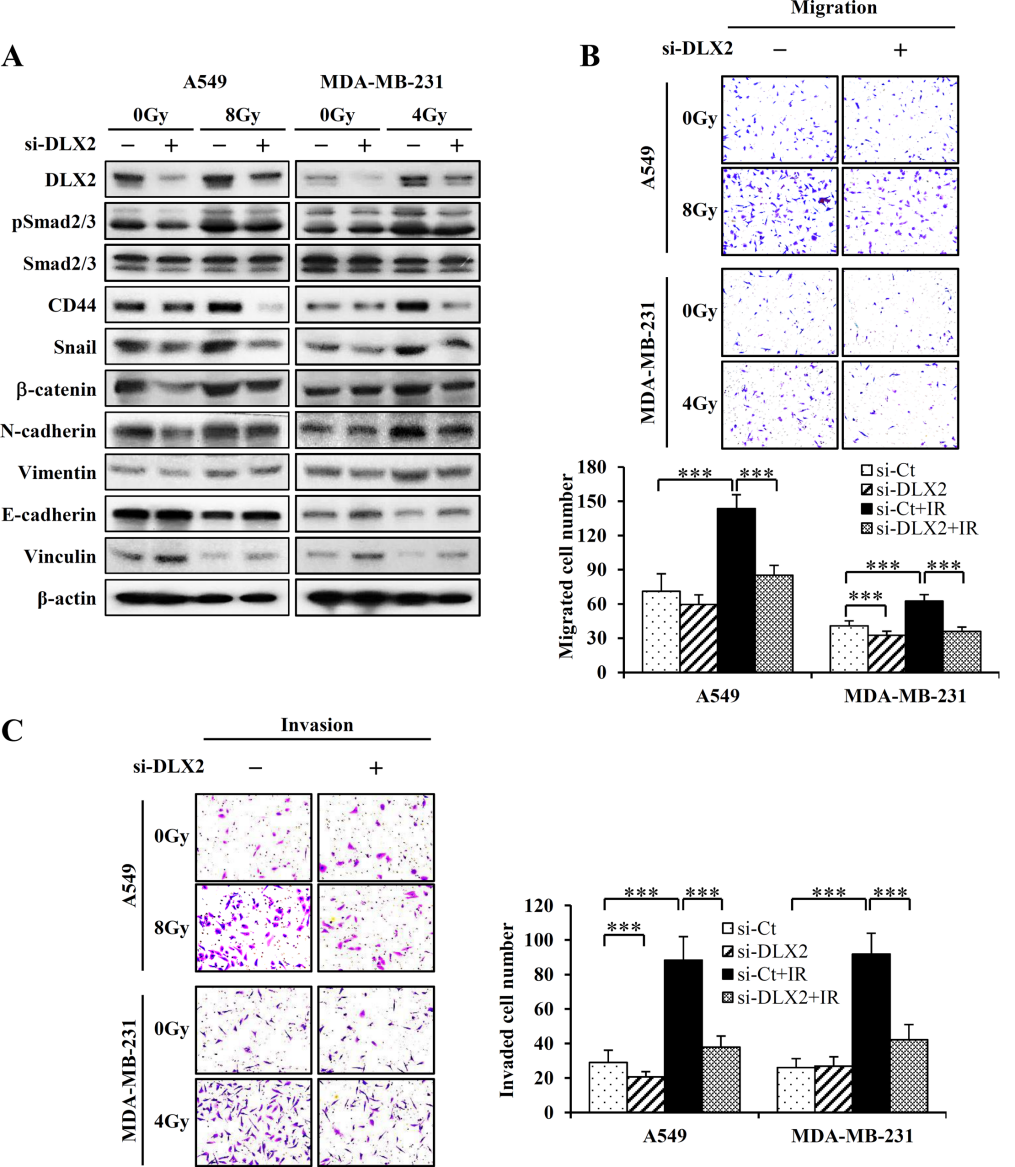
DLX2-silencing suppresses the expression of CSC markers, EMT and migratory and invasive ability in irradiated A549 and MDA-MB-231 cells. (A) Cells were transfected with 50 μM si-DLX2 or si-Ct and incubated at 37°C for 24 h, the cells were exposed to IR at 8Gy (A549) or 4Gy (MDA-MB-231) and incubated at 37°C for 24 h. Subsequently, cells were lysed and the lysates were subjected to western blot analysis. Two independent experiments obtained similar results. (B) Cells were transfected with 50 μM si-DLX2 or si-Ct and incubated at 37°C for 24 h. After cell detachment, the cells were exposed to IR at 8Gy (A549) or 4Gy (MDA-MB-231) and incubated at 37°C for 6 h (A549), 3 h (MDA-MB-231), in transwell (migration assay). The photographs are representative fields of migrated cells on the membrane. The graph indicates average of migrated cell number from three independent experiments ±SE (***P < 0.001). (C) Cells were transfected with 50 μM si-DLX2 or si-Ct and incubated at 37°C for 24 h. After cell detachment, the cells were exposed to IR at 8Gy (A549) or 4Gy (MDA-MB-231) and incubated at 37°C for 48 h (A549), 24 h (MDA-MB-231), in matrigel (invasion assay). The photographs are representative fields of invaded cells on the membrane. The graph indicates average of invaded cell number from three independent experiments ±SE (***P < 0.001).

Next, we examined whether DLX2 silencing would suppress metastatic potential and radioresistance conferred by IR. A549 and MDA-MB-231cells were transiently transfected with 50 μM siRNA of DLX2 (si-DLX2) or si-RNA control (si-Ct) for 24 h prior to irradiation (8Gy for A549, 4Gy for MDA-MB-231). Then we analyzed the expression of CSC and EMT related genes, migrating and invading abilities, and colony-forming ability. Silencing of DLX2 by siRNA prevented EMT as demonstrated by reduced protein levels of EMT positive markers (N-cadherin, Vimentin) (Fig 5A, S3 Fig and S4 Fig) and increased level of EMT negative markers (E-cadherin, Vinculin) in irradiated A549 and MDA-MB-231 cells (Fig 5A, S5 Fig and S6 Fig). Inhibition of DLX2 also prevented the induction of transcription factors critical for EMT (Snail and β-catenin), and a CSC marker (CD44) in irradiated A549 and MDA-MB-231 cells. Interestingly, activation of TGF-β signaling factor, Smad2/3, was not affected by si-DLX2 in irradiated cells (Fig 5A).

To investigate the anti-metastatic effect of si-DLX2 in irradiated cells, migration and invasion assays were performed. DLX2-silencing in MDA-MB-231 cells slightly reduced migration ability compare to the si-Ct cells. Also, in irradiated A549 and MDA-MB-231 cells, transfection of si-DLX2 decreased the migrating cell numbers by 1.7 folds compared to si-Ct transfection (Fig 5B). These results show that silencing of DLX2 by siRNA significantly inhibited cell motility of A549 and MDA-MB-231 cells. To examine whether cell invasion ability was similarly reduced by DLX2 silencing, irradiated cells were applied to invasion chambers and the numbers of adhesive cells were counted. In non-irradiated cells, DLX2-silencing inhibited invasive ability of A549 cells by 1.4 folds, but no significant change was observed in MDA-MB-231 cells. In irradiated A549 and MDA-MB-231 cells, transfection of si-DLX2 decreased invading cell numbers of A549 and MDA-MB-231 cells by 2.3 and 2.2 folds, respectively (Fig 5C). These results demonstrated that silencing of DLX2 in irradiated A549 and MDA-MB-231 cells significantly inhibited the expression of genes associated with CSC and EMT, and migratory and invasive ability which were induced by IR.

**Fig 6:**
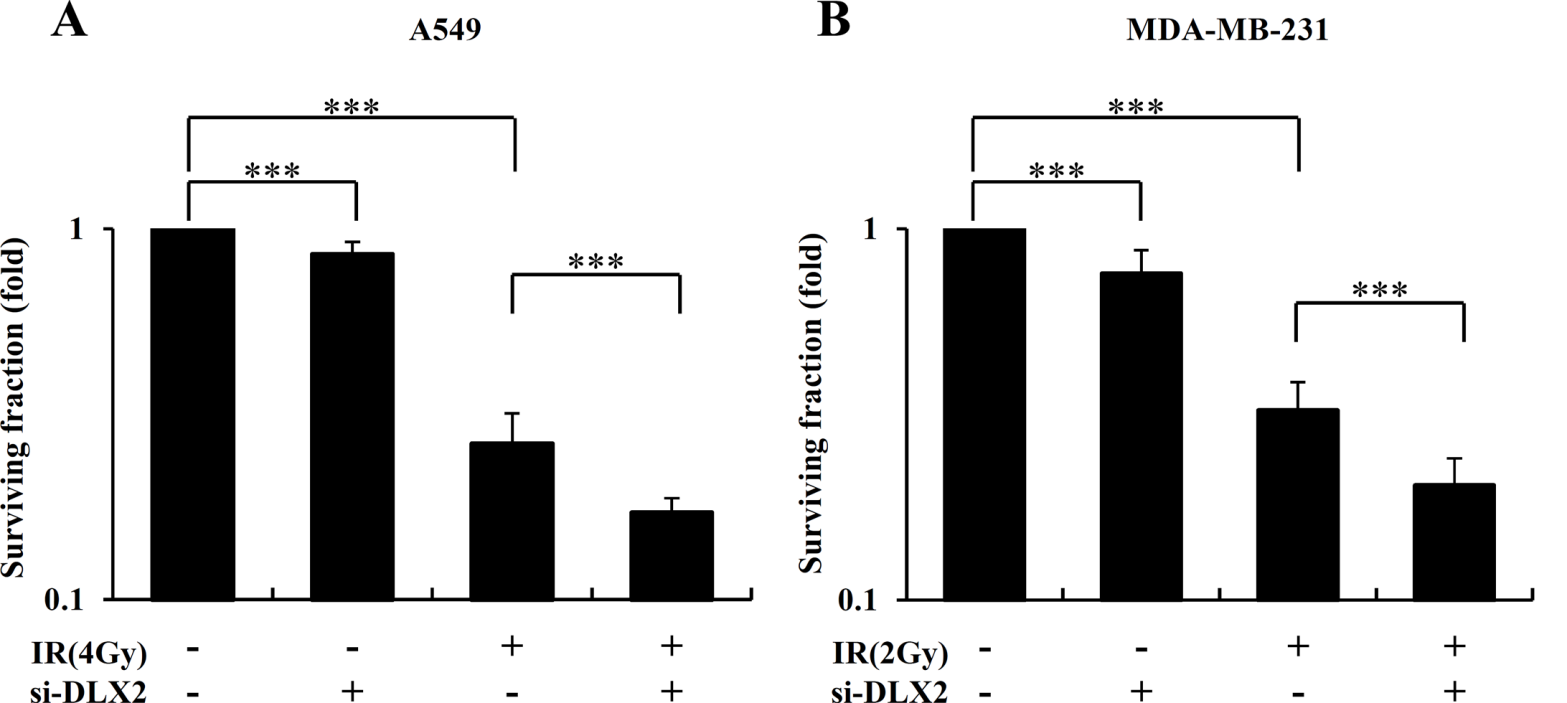
DLX2-silencing increases radiation sensitivity of A549 and MDA-MB-231 cells. Cells were transfected with 50 μM si-DLX2 or si-Ct and incubated at 37°C for 24 h. After cell detachment, the cells were exposed to IR at 4Gy (A549) or 2Gy (MDA-MB-231) and incubated at 37°C for 7 days (A549), 10 days (MDA-MB-231) and cell clonogenicity measured by clonogenic assay in A549 (A) and MDA-MB-231 (B). The photographs are representative plate of survival cells. The graph represents the average of three independent experiments ±SE (***P < 0.001).

We analyzed next whether DLX2-silencing influences cancer cell survival after irradiation. A549 and MDA-MB-231 cells transfected with si-DLX2 or si-Ct were irradiated at 4Gy and 2Gy, respectively, and then the colony formation was examined. In non-irradiated cells, DLX2 silencing resulted in a slight decrease of surviving fraction in both cell lines. In irradiated cells, DLX2 silencing leaded to an additional decrease in cell survival to a significant extent (1.5-fold decrease for A549 and 1.6-fold decrease for MDA-MB-231) (Fig 6A and 6B). These results indicated that silencing of DLX2 suppressed IR-induced EMT potential and enhanced radiation sensitivity.

### IR-induced DLX2 expression is regulated by Smad2/3

**Fig 7:**
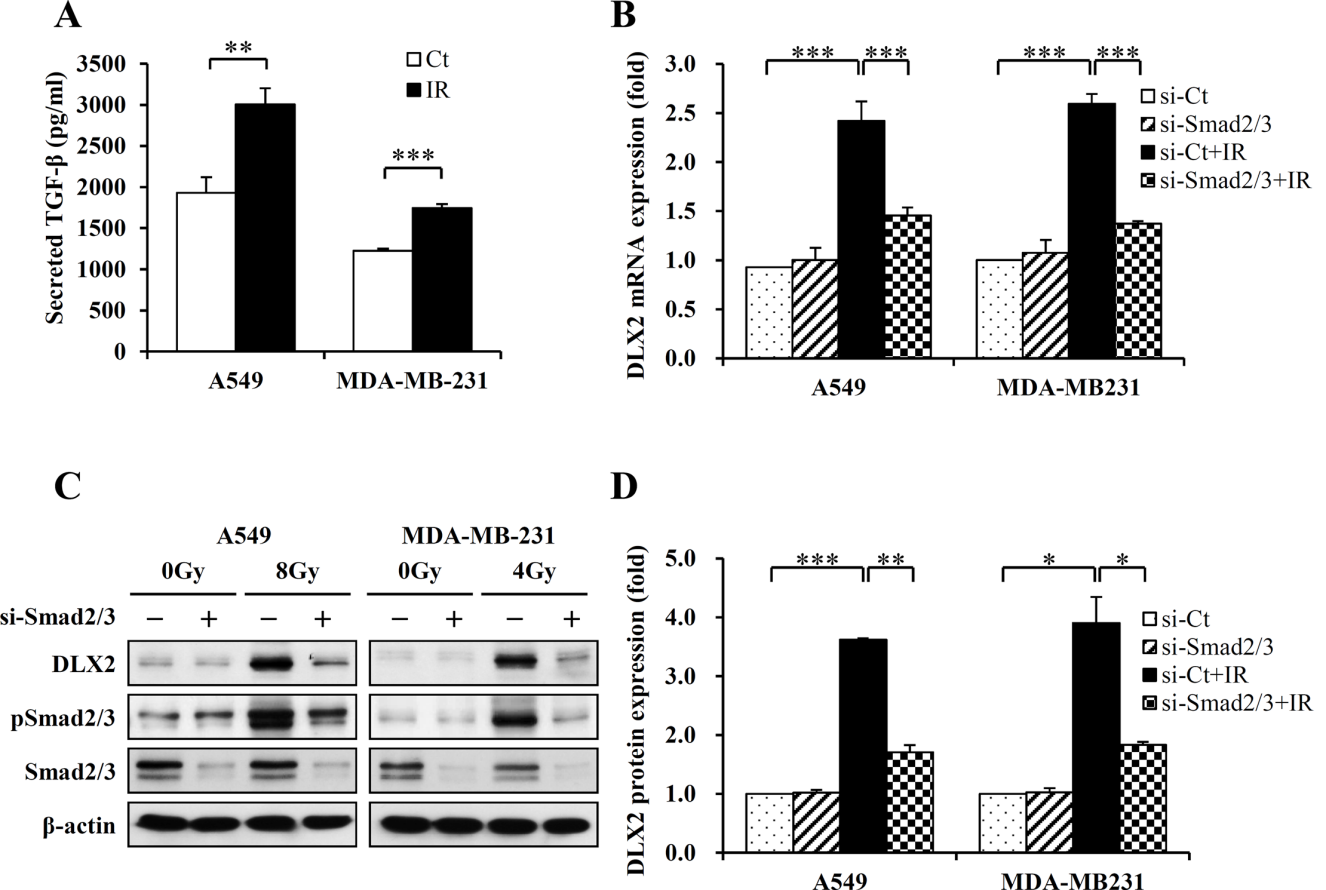
Silencing of Smad 2/3 abrogates the induction DLX2 in irradiated A549 and MDA-MB-231 cells. (A) The levels of immunoreactive TGF-β1 were quantified from the cell culture medium by ELISA, as described in the Materials and methods (***P < 0.001, **P < 0.01, versus Ct). Ct, control cell; IR, irradiated cells. (B) Cells were transfected with 100 μM si-Smad2/3 or si-Ct, incubated at 37°C for 24 h. Then the cells were exposed to IR at 8Gy (A549) or 4Gy (MDA-MB-231) and incubated at 37°C for 24 h. Total RNA was isolated from the cells and subjected to real time PCR analysis. The graph represents the average of three independent experiments ±SE (***P < 0.001). (C) Cells were transfected with 100 μM si-Smad2/3 or si-Ct and incubated at 37°C for 24 h. Then the cells were exposed to IR at 8Gy (A549) or 4Gy (MDA-MB-231) and incubated at 37°C for 24 h. Subsequently, the cell lysates were subjected to western blot analysis. Three independent experiments obtained similar results. (D) Protein levels were quantified by densitometry. Data are represented as relative values to those of si-Ct after normalization with β-actin (***P < 0.001, **P < 0.01, *P < 0.05 versus si-Ct).

IR promotes EMT via Smad-dependent TGF-β signaling in cancer cell lines [6, 27, 28], and DLX2 is a target gene of Smad-dependent TGF-β signaling and negative feedback factor [34]. Therefore, we examined the association of TGF-β signaling with IR-induced DLX2 expression. Irradiation of A549 cells (8Gy) and MDA-MB-231cells (4Gy) increased the amount of TGF-β1 secreted into the culture medium (Fig 7A). Also, IR promoted phosphorylation of TGF-β signaling factor Smad2/3 (Figs 2A, 2B, 3A, 5A and 7C, S1 Fig and S2 Fig). However, overexpression of DLX2 rather slightly decreased phosphorylation of Smad2/3 (Fig 3A). The phosphorylation of Smad2/3 was not affected either by si-DLX2 in irradiated A549 and MDA-MB-231 cells (Fig 5A). Therefore, we next examined whether the induction of DLX2 by IR is regulated by Smad2/3 signaling. Smad2/3 silencing by siRNA abrogated the IR-induced DLX2 mRNA expression (Fig 7B) and DLX2 protein expression (Fig 7C and 7D). These results indicate that IR-induced DLX2 expression is dependent on Smad2/3 signaling.

## Discussion

Radiation therapy is a critical component of cancer management, but some of surviving cancer cells gain radioresistance [1] and metastatic ability [2] on the repeated radiotherapy. The presence of cancer stem cells (CSC) among cancer cell population and the epithelial to mesenchymal transition (EMT) during repeated irradiation are thought to be important factors contributing to cancer relapse and metastasis [2]. To overcome this impediment, the identification and development of predictive biomarkers and targeted therapeutic strategy for CSC and EMT are especially important for prognosis evaluation and radiosensitivity improvement in radiation therapy.

The DLX2 genes is a member of the *Drosophila distal-less* family and has multiple functions as transcription factor in different stages of development or in different tissues and cell types [35]. According to recent reports, DLX2 deregulation is known to enhance cell survival and proliferation and prevent differentiation [36, 37]. Interestingly, Abnormal expression of DLX2 was found in malignant progression of human solid tumors including gastric adenocarcinoma, acute lymphoblastic leukemia, melanoma, glioma, breast, lung and prostate cancer [30, 32, 34, 38]. Also, DLX2 is speculated to be involved in tumor progression and aggressiveness by the regulation of metabolic stress-induced necrosis via the regulation of mitochondrial ROS [33].

These studies made us to focus on the potential role of DLX2 in the acquirement of CSC and EMT characteristics in IR-treated cancer cells. In this study, we have investigated the role of DLX2 in expression of CSCs and EMT-related genes, migration and invasion ability, radioresistance in irradiated A549 human lung cancer cells and MDA-MB-231 human breast cancer cells.

We first found that expression of DLX2 was increased by IR in A549 and MDA-MB-231 cells (Fig 2A and 2B). Besides, we confirmed the IR induced dose-and time-dependent changes of CSC marker (CD44), EMT positive markers (N-cadherin, Vimentin), transcription factors regulating EMT (Snail, β-catenin), and EMT negative markers (E-cadherin, Vinculin) in both cell lines (Fig 2A and 2B). By quantitative real-time PCR, we confirmed IR-induced upregulation of mRNA levels of stemness markers (OCT4, SOX2, LIF), transcription factors (Twist, Snail), and metastasis markers (MMP2, MMP7) in both cell lines (Fig 2C and 2D). Then we tested the effects of ectopic expression of DLX2 on the radiated A549 and MDA-MB-231 cells. We found that overexpression of DLX2 significantly increased the expression of CD44, N-cadherin, Vimentin and enhanced the migratory and invasive ability of A549 and MDA-MB-231 cells as similar as by IR (Fig 3A and 3B). In clonogeinic assay, DLX2-overexpressing cells showed significantly higher survival rate compared to vector-transfected cells after irradiation (Fig 4A and 4B). Conversely, down-regulation of DLX2 expression with si-DLX2 in irradiated A549 and MDA-MB-231 cells significantly inhibited the expression of genes associated with CSC and EMT, and migratory and invasive ability which were induced by IR (Fig 5A–5C). In clonogeinic assay, DLX2 silencing leaded to a significant decrease in cell survival in irradiated cells (Fig 6A and 6B). These results indicate that DLX2 expression promotes invasion, migration, and radioresistance of A549 and MDA-MB-231 cells.

Recent studies suggest that the stem-like properties of cancer cells may be quite plastic and associated with the EMT. In patients, EMT and CSCs increased resistance to radiotherapy [2]. EMT is promoted by various soluble factors, and especially TGF-β is a strong inducer for EMT [26]. In TGFβ-induced EMT process, cells lead dynamic cytoskeletal remodeling and morphological change of epithelial to mesenchymal transition [26, 29, 39]. Importantly, several reports indicated that IR induces Smad-dependent activation of TGF-β signaling in cancer [40–42], and the blocking of TGF-β pathway prior to irradiation increased radiosensitivity of murine and human lung cancer cells [43]. Interestingly, DLX2 is reported to play a dual role in TGF-β signaling [34]. DLX2 is a downstream target gene of phosphorylated Smad2/3 and upregulated upon TGF-β treatment. On the other hand, DLX2 can also act as a negative feedback factor of TGF-β signaling and inhibit TGF-β-induced cell-cycle arrest and apoptosis increasing primary tumor growth and metastasis in B16 melanoma cells. In spite of these previous reports, the role of DLX2 in acquisition of CSC and EMT characteristics and its association with Smad-dependent TGF-β signaling in irradiated cancer cells have been remained elusive. In this study, we demonstrated that DLX2 acted as a crucial downstream mediator of TGF-β signaling in irradiated A549 and MDA-MB-231 cells. We observed that IR increased the phosphorylation of Smad2/3, a TGF-β signaling factor (Fig 2A and 2B). However, phosphorylation of Smad2/3 was not affected either by overexpression of DLX2 (Fig 3A) or by silencing of DLX2 (Fig 5A). We further investigated whether the induction of DLX2 by IR is regulated by Smad2/3 signaling and found that Smad2/3 silencing by siRNA abrogated the IR-induced DLX2 expression (Fig 7). These results indicated that DLX2 is a downstream target gene of phosphorylated Smad2/3, and IR-induced DLX2 expression is dependent on Smad2/3 signaling. Although a vast of reports support that TGF-β is a key regulator of EMT and Smads mediate this process, a few studies reported that TGF-β-induced EMT process can also occur through Smad-independent pathways [44, 45]. Therefore, the role of DLX2 in EMT process may differ with respect to the types of tumor cells and EMT stimuli.

In this study, we showed for the first time that DLX2 is associated with IR-induced EMT process and acquisition of CSC properties. Smad2/3 was activated in response to IR and induced DLX2 expression. DLX2 in turn promoted the expression of CSC and EMT-related genes resulting in the enhanced migration and invasion ability and radioresistance in A549 and MDA-MB-231 human cancer cells. Since elevated expression of DLX2 has been found in many malignant tumors [30, 32, 34, 38], our results strongly support the possible involvement of DLX2 in EMT and radioresistance in many tumors. However, the role of DLX2 should be further confirmed in more in vitro studies with other human tumor cells as well as in animal studies to utilize the DLX2 as a therapeutic target for reducing metastatic ability and increasing radiosensitivity of tumors.

## Supporting Information

S1 Fig Radiation dose-dependent protein expression of DLX2, CSC and EMT markers in A549 and MDA-MB-231 cells. A549 (A) and MDA-MB-231(B) cells were exposed to IR at 0-8Gy and incubated at 37°C for 24 h. Lysates were subjected to western blot analysis. Two independent experiments obtained similar results (Fig 2A). Protein levels were quantified by densitometry. Data are represented as relative values to those of si-Ct after normalization with β-actin (***P < 0.001, **P < 0.01, *P < 0.05 versus 0Gy).
(TIF)

S2 Fig Time-dependent protein expression of DLX2, CSC and EMT markers in irradiated A549 and MDA-MB-231 cells. A549 (A) and MDA-MB-231 (B) cells were harvested on 0/3/6/24 h after 8Gy (A549) or 4Gy (MDA-MB-231). Lysates were subjected to western blot analysis with the labeled antibodies. The β-actin was used as a loading control. Two independent experiments obtained similar results (Fig 2B). Protein levels were quantified by densitometry. Data are represented as relative values to those of si-Ct after normalization with β-actin (***P < 0.001, **P < 0.01, *P < 0.05 versus 0 h).
(TIF)

S3 Fig DLX2-silencing suppresses IR-induced expression of N-cadherin in A549 and MDA-MB-231 cells in immunofluorescence staining. A549 (A) and MDA-MB-231(B) cells were transfected with si-Ct or si-DLX2 for 24 h and then incubated for 24 h after IR. Focal adhesions were visualized by immunofluorescence staining of F-actin stress fibers with phalloidin (green, a, b and c) and N-cadherin (red, d, e and f). The nucleus is stained with DAPI (g, h and i). (j, k and l) Merged images. The expression of stress fibers and N-cadherin is increased during IR stimulation (a/b, d/e). Also, DLX2-silencing suppresses the expression of IR-induced stress fiber and N-cadherin (b/c, e/f). The magnificent of the image is ×100.
(TIF)

S4 Fig DLX2-silencing suppresses IR-induced expression of Vimentin in A549 and MDA-MB-231 cells in immunofluorescence staining. A549 (A) and MDA-MB-231(B) cells were transfected with si-Ct or si-DLX2 for 24 h and then incubated for 24 h after IR. Focal adhesions were visualized by immunofluorescence staining of F-actin stress fibers with phalloidin (green, a, b and c) and Vimentin (red, d, e and f). The nucleus is stained with DAPI (g, h and i). (j, k and l) Merged images. The expression of stress fibers and Vimentin is increased during IR stimulation (a/b, d/e). Also, DLX2-silencing suppresses the expression of IR-induced stress fiber and Vimentin (b/c, e/f). The magnificent of the image is ×100.
(TIF)

S5 Fig DLX2-silencing restores IR-suppressed expression of E-cadherin in A549 and MDA-MB-231 cells in immunofluorescence staining. A549 (A) and MDA-MB-231(B) cells were transfected with si-Ct or si-DLX2 for 24 h and then incubated for 24 h after IR. Focal adhesions were visualized by immunofluorescence staining of F-actin stress fibers with phalloidin (green, a, b and c) and E-cadherin (red, d, e and f). The nucleus is stained with DAPI (g, h and i). (j, k and l) Merged images. The expression of stress fibers is increased and the expression (a/b) of E-cadherin is decreased during IR stimulation (d/e). Also, DLX2-silencing repairs the expression of IR-inhibited E-cadherin (e/f). The magnificent of the image is ×100.
(TIF)

S6 Fig DLX2-silencing restores IR-suppressed expression of Vinculin in A549 and MDA-MB-231 cells in immunofluorescence staining. A549 (A) and MDA-MB-231(B) cells were transfected with si-Ct or si-DLX2 for 24 h and then incubated for 24 h after IR. Focal adhesions were visualized by immunofluorescence staining of F-actin stress fibers with phalloidin (green, a, b and c) and Vinculin (red, d, e and f). The nucleus is stained with DAPI (g, h and i). (j, k and l) Merged images. The expression of stress fibers is increased and the expression (a/b) of Vinculin is decreased during IR stimulation (d/e). Also, DLX2-silencing repairs the expression of IR-inhibited Vinculin (e/f). The magnificent of the image is ×100.
(TIF)
