# Supplementary figures and images for: Smad2/3-Regulated Expression of DLX2 Is Associated with Radiation-Induced Epithelial-Mesenchymal Transition and Radioresistance of A549 and MDA-MB-231 Human Cancer Cell Lines
Source: PLoS One. 2016 Jan 22;11(1):e0147343. doi: 10.1371/journal.pone.0147343 (PMC4723265; doi:10.1371/journal.pone.0147343)

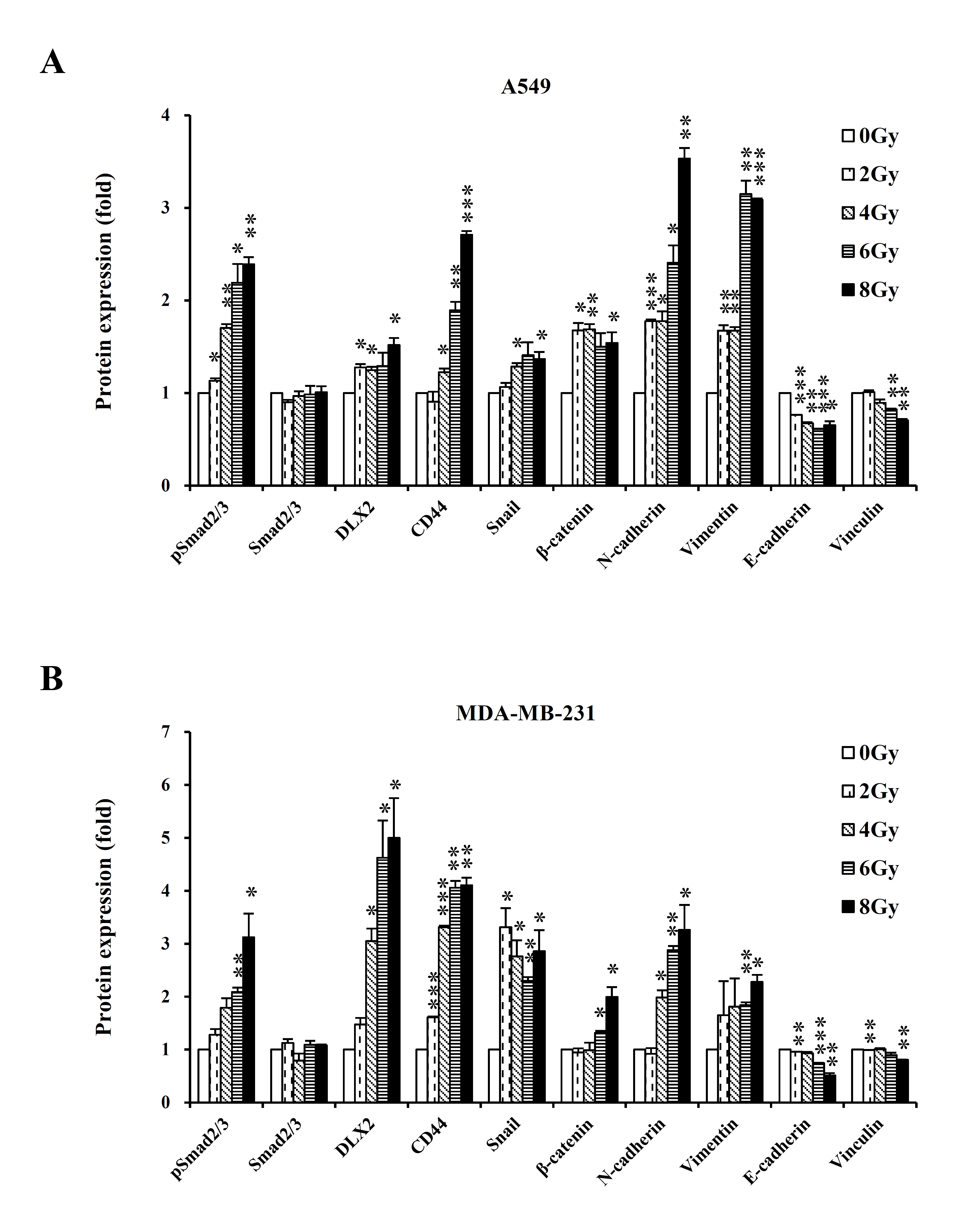

Supplement: S1 Fig — A549 (A) and MDA-MB-231(B) cells were exposed to IR at 0-8Gy and incubated at 37°C for 24 h. Lysates were subjected to western blot analysis. Two independent experiments obtained similar results (Fig 2A). Protein levels were quantified by densitometry. Data are represented as relative values to those of si-Ct after normalization with β-actin (***P < 0.001, **P < 0.01, *P < 0.05 versus 0Gy). (TIF) [file pone.0147343.s001.tif]

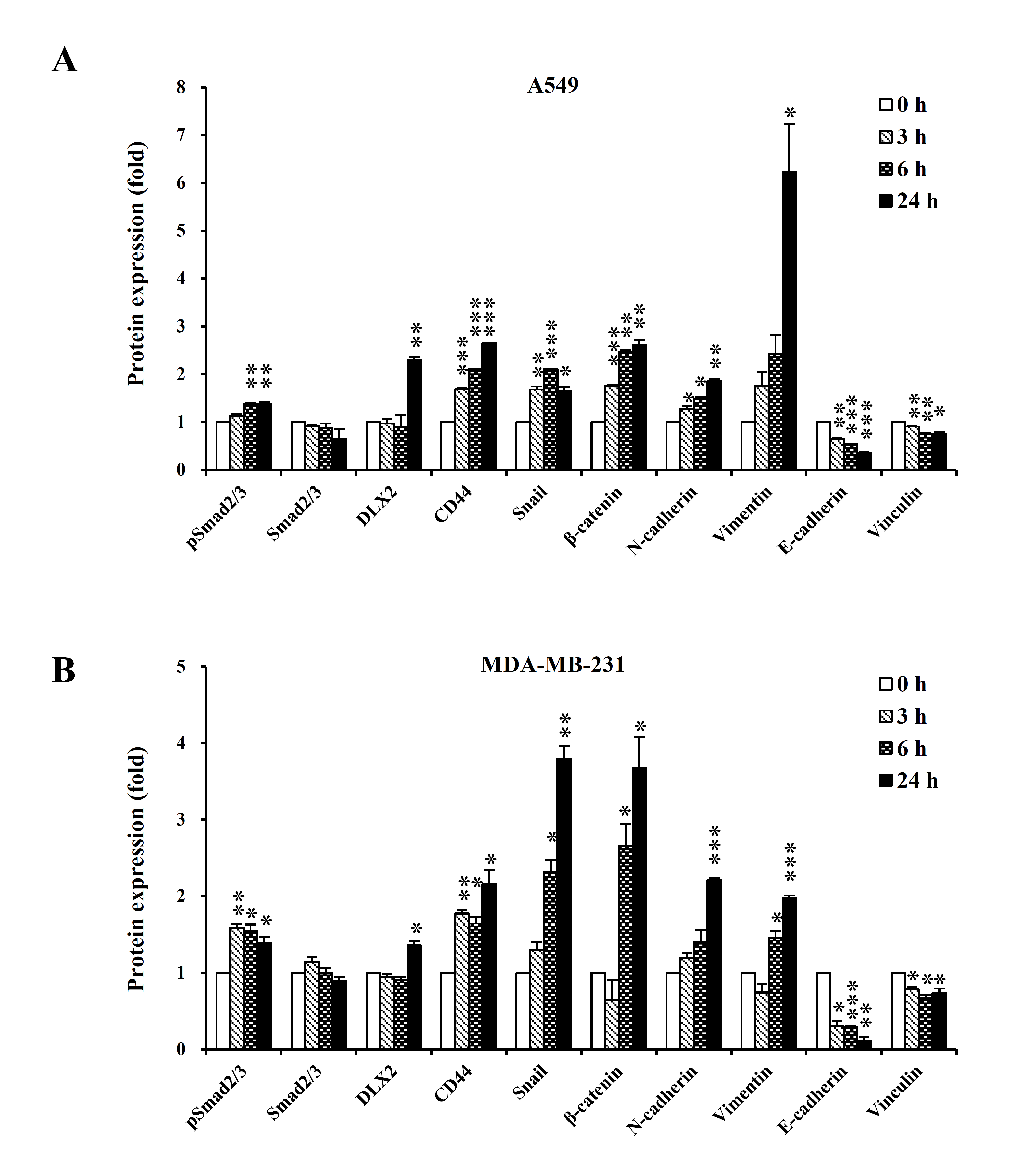

Supplement: S2 Fig — A549 (A) and MDA-MB-231 (B) cells were harvested on 0/3/6/24 h after 8Gy (A549) or 4Gy (MDA-MB-231). Lysates were subjected to western blot analysis with the labeled antibodies. The β-actin was used as a loading control. Two independent experiments obtained similar results (Fig 2B). Protein levels were quantified by densitometry. Data are represented as relative values to those of si-Ct after normalization with β-actin (***P < 0.001, **P < 0.01, *P < 0.05 versus 0 h). (TIF) [file pone.0147343.s002.tif]

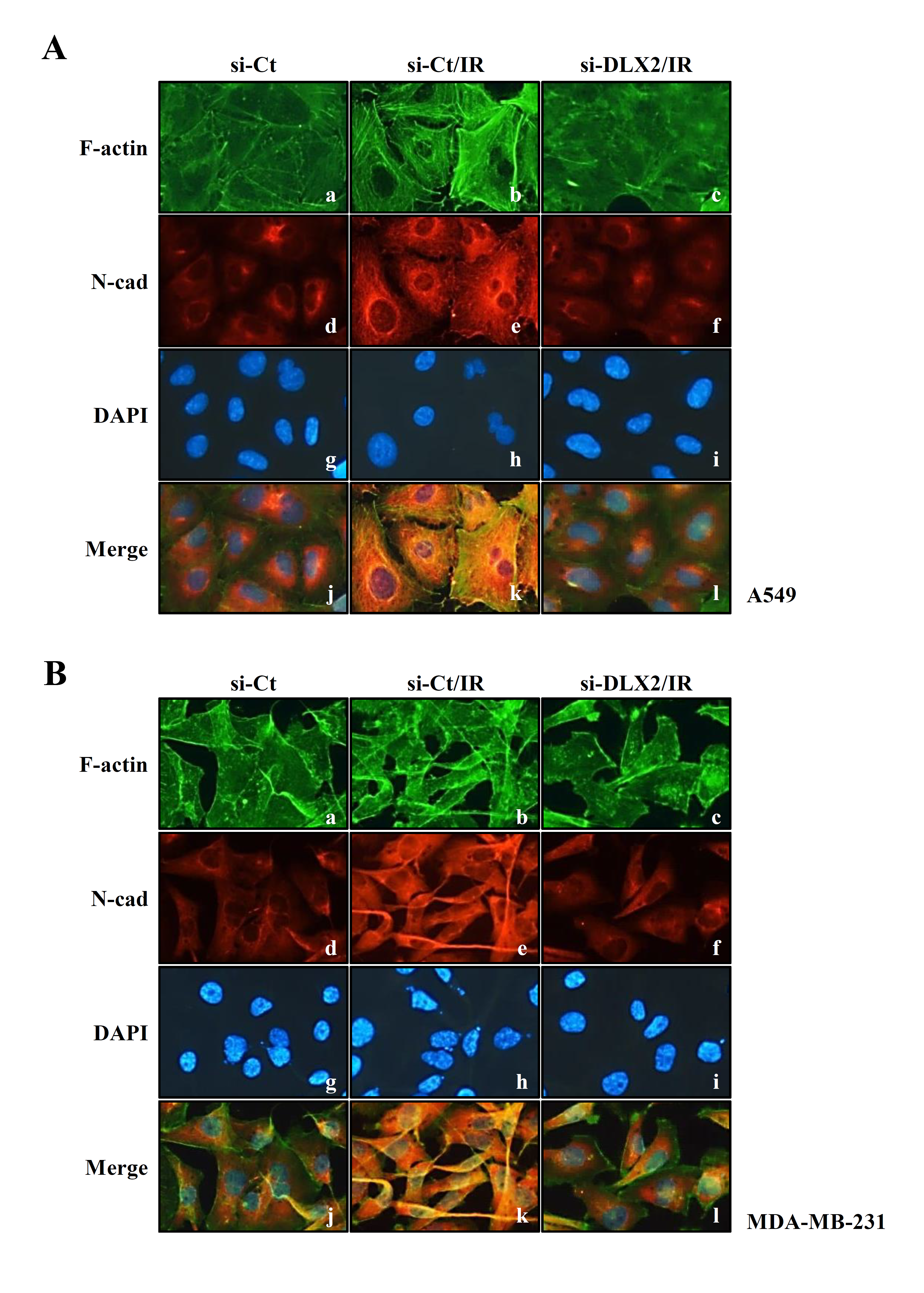

Supplement: S3 Fig — A549 (A) and MDA-MB-231(B) cells were transfected with si-Ct or si-DLX2 for 24 h and then incubated for 24 h after IR. Focal adhesions were visualized by immunofluorescence staining of F-actin stress fibers with phalloidin (green, a, b and c) and N-cadherin (red, d, e and f). The nucleus is stained with DAPI (g, h and i). (j, k and l) Merged images. The expression of stress fibers and N-cadherin is increased during IR stimulation (a/b, d/e). Also, DLX2-silencing suppresses the expression of IR-induced stress fiber and N-cadherin (b/c, e/f). The magnificent of the image is ×100. (TIF) [file pone.0147343.s003.tif]

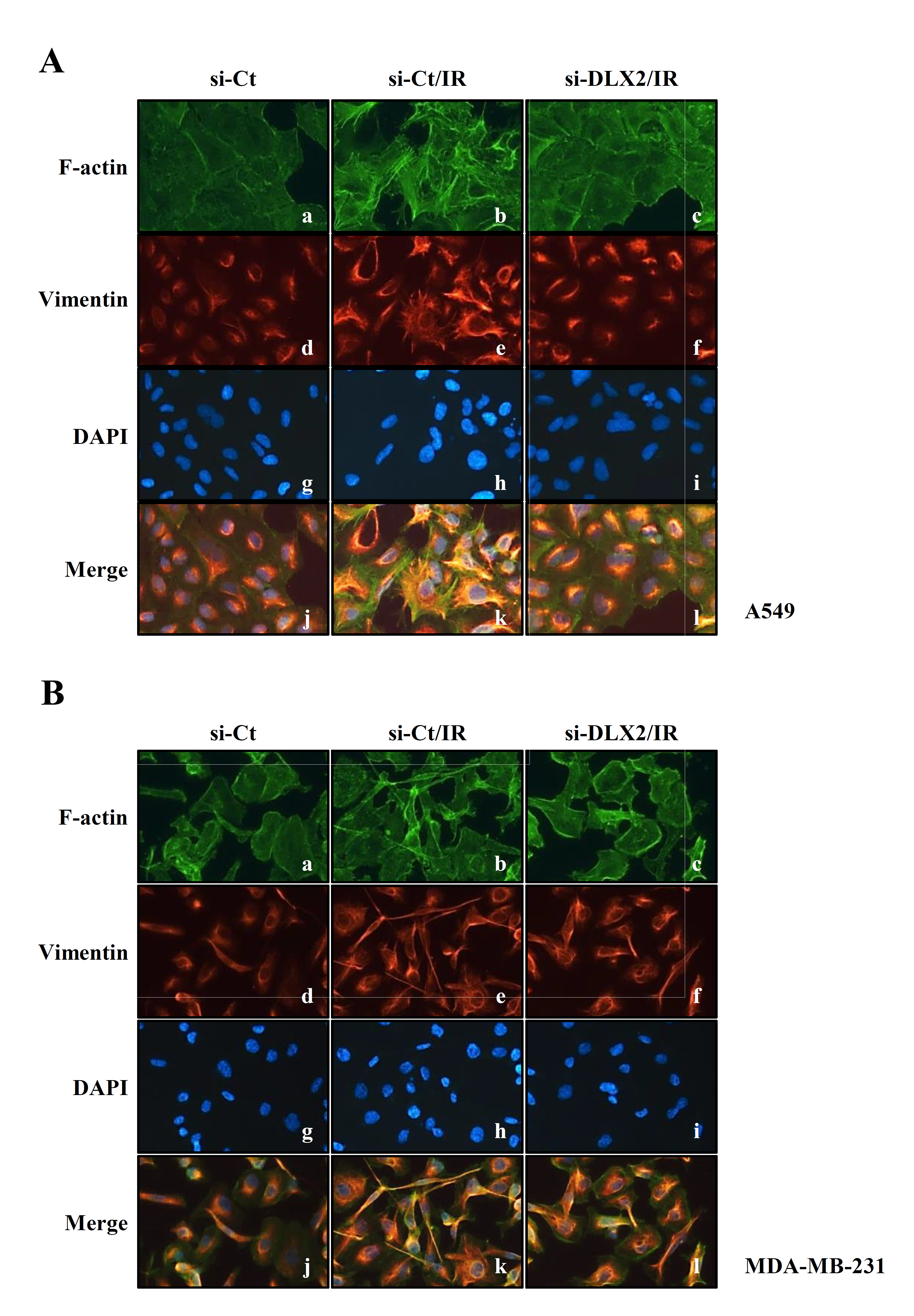

Supplement: S4 Fig — A549 (A) and MDA-MB-231(B) cells were transfected with si-Ct or si-DLX2 for 24 h and then incubated for 24 h after IR. Focal adhesions were visualized by immunofluorescence staining of F-actin stress fibers with phalloidin (green, a, b and c) and Vimentin (red, d, e and f). The nucleus is stained with DAPI (g, h and i). (j, k and l) Merged images. The expression of stress fibers and Vimentin is increased during IR stimulation (a/b, d/e). Also, DLX2-silencing suppresses the expression of IR-induced stress fiber and Vimentin (b/c, e/f). The magnificent of the image is ×100. (TIF) [file pone.0147343.s004.tif]

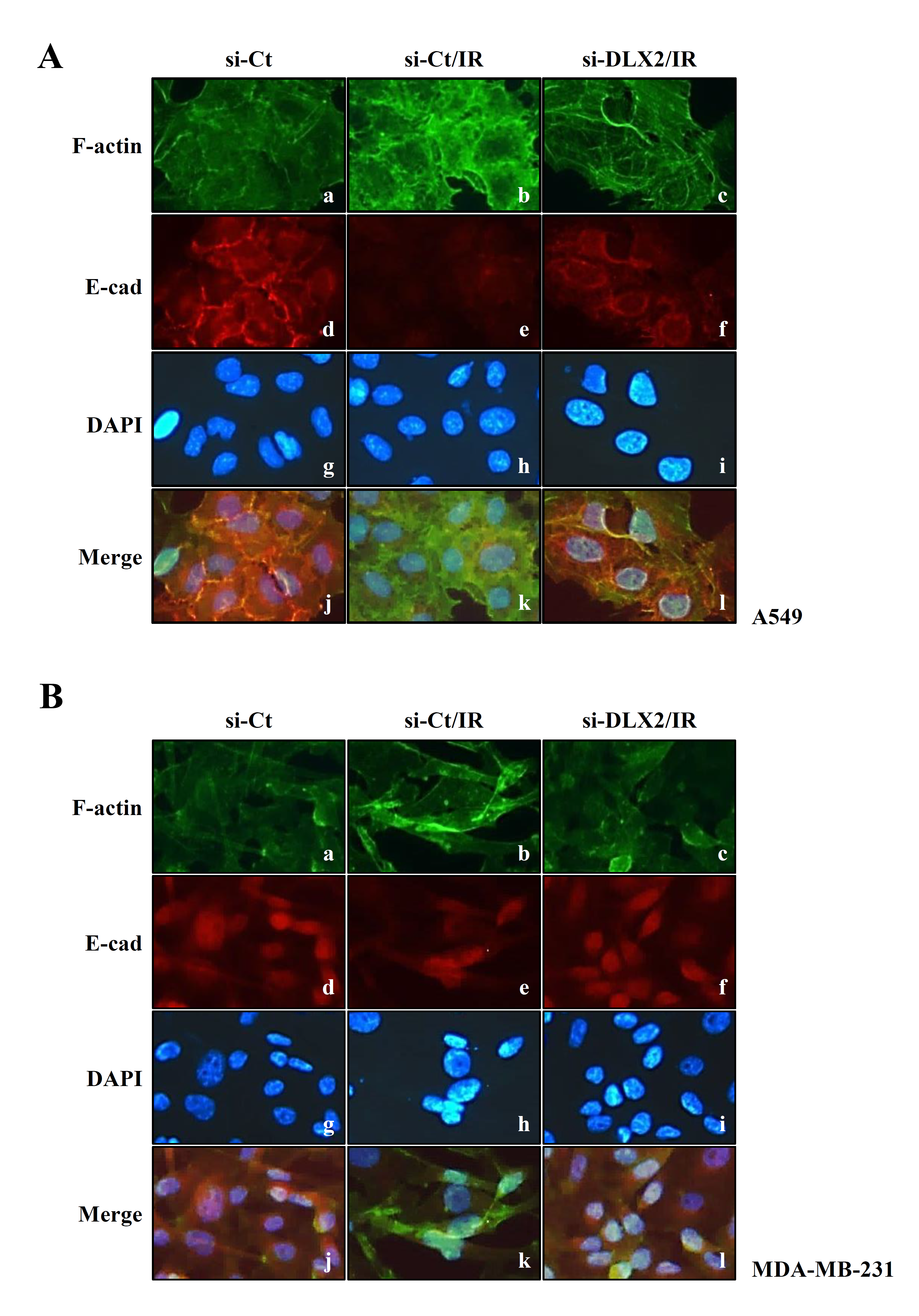

Supplement: S5 Fig — A549 (A) and MDA-MB-231(B) cells were transfected with si-Ct or si-DLX2 for 24 h and then incubated for 24 h after IR. Focal adhesions were visualized by immunofluorescence staining of F-actin stress fibers with phalloidin (green, a, b and c) and E-cadherin (red, d, e and f). The nucleus is stained with DAPI (g, h and i). (j, k and l) Merged images. The expression of stress fibers is increased and the expression (a/b) of E-cadherin is decreased during IR stimulation (d/e). Also, DLX2-silencing repairs the expression of IR-inhibited E-cadherin (e/f). The magnificent of the image is ×100. (TIF) [file pone.0147343.s005.tif]

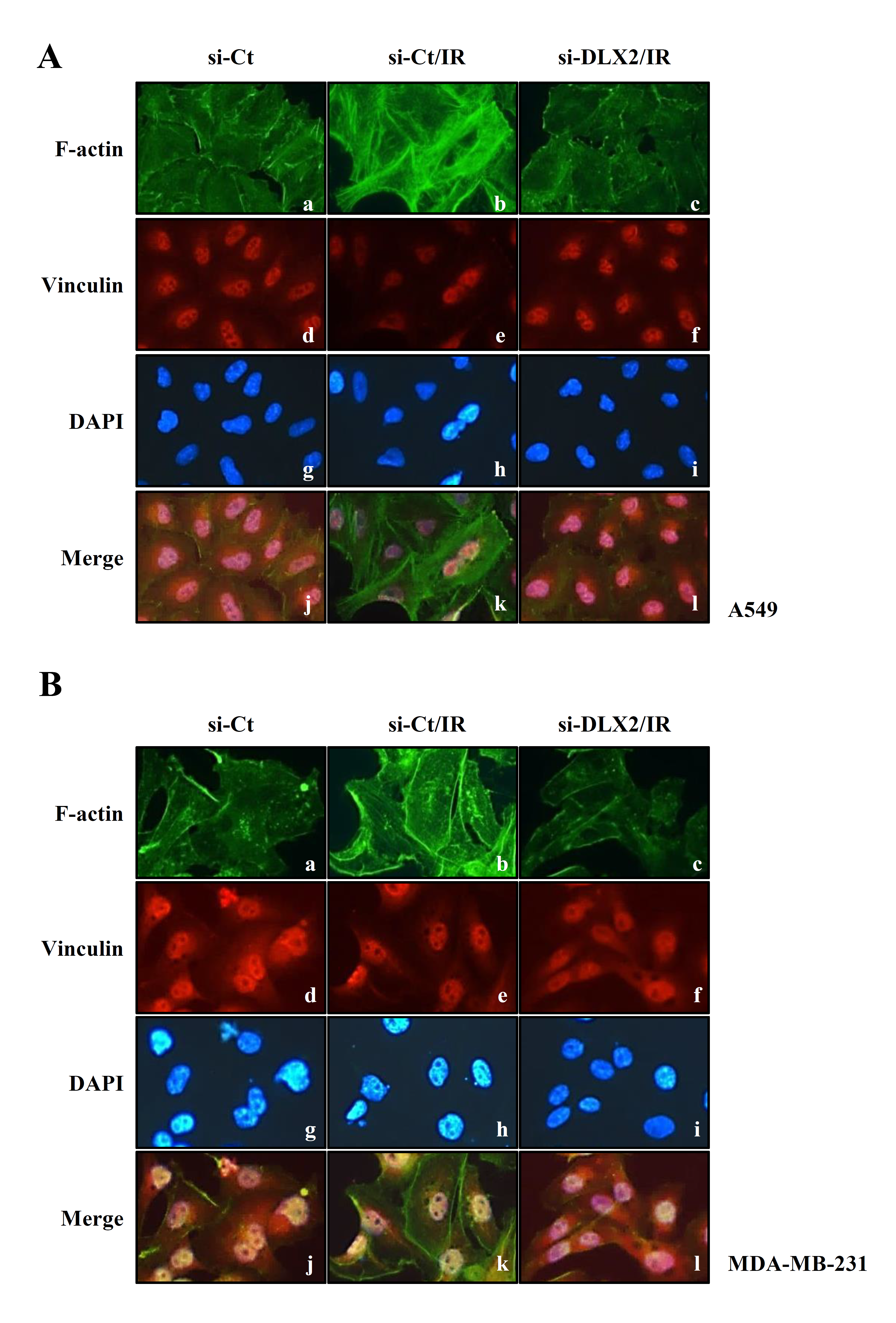

Supplement: S6 Fig — A549 (A) and MDA-MB-231(B) cells were transfected with si-Ct or si-DLX2 for 24 h and then incubated for 24 h after IR. Focal adhesions were visualized by immunofluorescence staining of F-actin stress fibers with phalloidin (green, a, b and c) and Vinculin (red, d, e and f). The nucleus is stained with DAPI (g, h and i). (j, k and l) Merged images. The expression of stress fibers is increased and the expression (a/b) of Vinculin is decreased during IR stimulation (d/e). Also, DLX2-silencing repairs the expression of IR-inhibited Vinculin (e/f). The magnificent of the image is ×100. (TIF) [file pone.0147343.s006.tif]
